# A302 NON-INVASIVE BIOMARKERS PREDICTING THE PROGRESSION OF MASLD TO ADVANCED FIBROSIS: A PROSPECTIVE COHORT STUDY

**DOI:** 10.1093/jcag/gwad061.302

**Published:** 2024-02-14

**Authors:** Y Song, R Mortuza, J Arab, M Khan

**Affiliations:** Division of Gastroenterology, Western University, London, ON, Canada; Division of Gastroenterology, Western University, London, ON, Canada; Division of Gastroenterology, Western University, London, ON, Canada; Division of Gastroenterology, Western University, London, ON, Canada

## Abstract

**Background:**

Metabolic dysfunction-associated steatotic liver disease (MASLD) affects around 30% of the global population and is one of the most prevalent non-communicable diseases. Due to its high prevalence, it is becoming the most common cause of liver-related mortality in all populations worldwide. Current standards of care for the treatment of MASLD encompass mostly lifestyle measures that are nonspecific and difficult for clinicians to ensure compliance. Although not all patients with MASLD progress to more advanced disease, the high prevalence of MASLD in the general population means the absolute number of “at risk” individuals are substantial and places a significant burden on the healthcare system. As a result, MASLD is currently viewed as a heterogeneous disease with varying rates of disease progression.

**Aims:**

We aim to elucidate the predictors of disease progression in MASLD to develop and guide interventions while the disease is reversible.

**Methods:**

Prospective chart review on patients seen in MASLD clinic at LHSC on CERNER from Sept. 2021 – Apr. 30, 2023. A REDCap database was constructed pertaining to a) basic patient information b) basic medical history c) patient lifestyle habits d) InBody bioimpedance analysis if available f) laboratory data g) elastography, including liver stiffness measurement (LSM) and controlled attenuation parameter (CAP). Collected data were exported into a spreadsheet to allow for analysis. IBM SPSS was used to analyze descriptive statistics and regression analyses.

**Results:**

A total of 116 encounters were reviewed, including both enrolment and follow-ups. HbA1c percent is positively correlated with LSM (r=0.28, pampersand:003C0.05). The longer that the patient has been diagnosed with NAFLD (current age minus age of initial diagnosis), the more likely the patient has higher LSM scores (r= 0.23, pampersand:003C0.05). Platelet levels are negatively correlated with LSM (r=-0.28, pampersand:003C0.05). There is no difference between cannabis users and non-users in the degree of CAP or LSM. Only n=15 InBody bioimpedance analyses were included; preliminary results showed Trunk Reactance at 250kHz is correlated with LSM with (r=0.593 pampersand:003C0.05).

**Conclusions:**

Patients with higher HbA1c, lower platelets and longer duration of disease are at higher risk for developing advanced fibrosis. This can allow the development of a better predictive model to find F2-F3 patients without using FibroScan or a liver biopsy, which is helpful in the primary care setting, given the prevalence of MASLD. InBody bioimpedance studies are a useful tool to predict LSM, although more data is needed to ensure the correlation is sufficiently powered. Phenotyping patients would lead to more accurate prognostication, facilitate better monitoring to prevent decompensation and allow for the development of precision-based, personalized management plans.

**Funding Agencies:**

None

